# Epidemiological characteristics and transmission dynamics of the COVID-19 outbreak in Hohhot, China: a time-varying SQEIAHR model analysis

**DOI:** 10.3389/fpubh.2023.1175869

**Published:** 2023-06-21

**Authors:** Yifei Ma, Shujun Xu, Yuxin Luo, Yao Qin, Jiantao Li, Lijian Lei, Lu He, Tong Wang, Hongmei Yu, Jun Xie

**Affiliations:** ^1^School of Public Health, Shanxi Medical University, Taiyuan, China; ^2^School of Management, Shanxi Medical University, Taiyuan, China; ^3^Shanxi Provincial Key Laboratory of Major Diseases Risk Assessment, Taiyuan, China; ^4^Center of Reverse Microbial Etiology, Shanxi Medical University, Taiyuan, China

**Keywords:** COVID-19, epidemiological characteristics, transmission dynamics, time-varying SQEIAHR model, effective reproduction number, higher stringency measures

## Abstract

**Background:**

On September 28, 2022, the first case of Omicron subvariant BF.7 was discovered among coronavirus disease 2019 (COVID-19) infections in Hohhot, China, and then the epidemic broke out on a large scale during the National Day holiday. It is imminently necessary to construct a mathematical model to investigate the transmission dynamics of COVID-19 in Hohhot.

**Methods:**

In this study, we first investigated the epidemiological characteristics of COVID-19 cases in Hohhot, including the spatiotemporal distribution and sociodemographic distribution. Then, we proposed a time-varying Susceptible-Quarantined Susceptible-Exposed-Quarantined Exposed-Infected-Asymptomatic-Hospitalized-Removed (SQEIAHR) model to derive the epidemic curves. The next-generation matrix method was used to calculate the effective reproduction number (*R*_e_). Finally, we explored the effects of higher stringency measures on the development of the epidemic through scenario analysis.

**Results:**

Of the 4,889 positive infected cases, the vast majority were asymptomatic and mild, mainly concentrated in central areas such as Xincheng District. People in the 30–59 age group primarily were affected by the current outbreak, accounting for 53.74%, but females and males were almost equally affected (1.03:1). Community screening (35.70%) and centralized isolation screening (26.28%) were the main ways to identify positive infected cases. Our model predicted the peak of the epidemic on October 6, 2022, the dynamic zero-COVID date on October 15, 2022, a number of peak cases of 629, and a cumulative number of infections of 4,963 (95% confidential interval (95%CI): 4,692 ~ 5,267), all four of which were highly consistent with the actual situation in Hohhot. Early in the outbreak, the basic reproduction number (*R*_0_) was approximately 7.01 (95%CI: 6.93 ~ 7.09), and then *R*_e_ declined sharply to below 1.0 on October 6, 2022. Scenario analysis of higher stringency measures showed the importance of decreasing the transmission rate and increasing the quarantine rate to shorten the time to peak, dynamic zero-COVID and an *R*_e_ below 1.0, as well as to reduce the number of peak cases and final affected population.

**Conclusion:**

Our model was effective in predicting the epidemic trends of COVID-19, and the implementation of a more stringent combination of measures was indispensable in containing the spread of the virus.

## Introduction

As the most widespread and severe public health crisis in the last 100 years, coronavirus disease 2019 (COVID-19) has affected more than 200 countries to varying degrees (1, 2), with 600 million people infected and 6.5 million deaths to date. At present, Omicron has replaced Delta as the predominant strain worldwide, accounting for approximately 58.5% ~ 80.6% of cases (3). From March 1 to April 22, 2022, more than half a million local Omicron cases were reported in almost all provinces across China (4). In the UK, the prevalence of the Omicron variant and its multiple subvariants has led to high infection rates across all age groups, particularly among young children (5). The monthly incidence rate of COVID-19 infections (new cases per 1,000 persons per day) in the US increased substantially when Omicron became the dominant strain compared to Delta (3.8–5.2 vs. 0.5–0.7) (6). The sheer number of Omicron cases has strained health care systems worldwide.

Recent outbreaks of COVID-19 in mainland China were distributed in multiple locations, with wide coverage and frequent occurrences, involving many provinces, cities, counties and districts nationwide, so the epidemic prevention and control situation remains severe and complex. On September 28, 2022, one new indigenous confirmed case of COVID-19 was reported in Hohhot, Inner Mongolia Autonomous Region. This was the first time that the Omicron subvariant BF.7 had appeared in China. The variant, called BA.5.2.1.7 or BF.7 for short, is an offshoot of the Omicron subvariant BA.5, which has the characteristics of extremely strong transmissibility, pathogenicity and immune escape capacity. As of October 3, 2022, the cumulative number of COVID-19 cases reported in Hohhot has exceeded 500, suggesting that the anti-epidemic situation is not optimistic. A serious concern at present is that there is no clear description or consensus on the epidemiological characteristics and future course of the current outbreak. There is an urgent need to explore the epidemiological characteristics of COVID-19 in Hohhot and to develop a prediction model to estimate the incidence trend and determine the priorities of prevention and control measures to provide a scientific basis for an effective response to subsequent outbreaks.

Since the outbreak of COVID-19, scholars have carried out related research from various perspectives, including mathematical modeling, epidemiology, and spatial analysis. Infectious disease dynamic models are regarded as important tools to forecast the prevalence of COVID-19, among which Susceptible-Infected-Removed (SIR) and Susceptible-Exposed-Infected-Removed (SEIR) compartmental models are particularly popular (7–9). Huarachi Olivera RE et al. applied the SIR model to characterize the epidemic evolution of COVID-19 and found that stringent measures can effectively prevent the spread of COVID-19 (10). Hao X et al. used the SAPHIRE model to reconstruct the full-spectrum dynamics of COVID-19 (11). Cai J et al. developed an age-structured stochastic compartmental SLIRL model to project the COVID-19 burden under mitigation scenarios (4). Shin HY conducted multi-stage estimations of the COVID-19 transmission dynamics using SEIR(D) epidemic models, and the results showed that the SEIR(D) is useful and informative (12). Kuniya T estimated the basic reproduction number (*R*_0_) for the epidemic in Japan based on the SEIR compartmental model using a least-square-based method with Poisson noise (13). A Bayesian approach has also been proposed to monitor the COVID-19 pandemic and to estimate the proportion of people who died or became infected with SARS-CoV-2 (14). Some studies have investigated the epidemiology and spatial distribution of COVID-19, for example, examining the associations of self-reported COVID-19 infection and SARS-CoV-2 serology test results with persistent physical symptoms and analyzing the spatiotemporal variations of cases and deaths of COVID-19 (15, 16).

Although the above models have achieved rather good prediction results, the SEIR model and its parameters still need to be further modified in due course because the transmission characteristics of the mutant strains differ considerably from those of past strains, and the speed and intensity of implementation of prevention and control measures are inconsistent across regions. Our objectives were to (1) establish a dynamic model based on the current epidemiological situation in Hohhot, (2) predict the development trends of COVID-19, including the peak, size, and reproduction number, and (3) evaluate the effectiveness and priority of different non-pharmaceutical interventions in halting the spread of COVID-19.

## Methods

### Data sources

Daily COVID-19 report data were obtained from the Inner Mongolia Autonomous Region Health and Health Commission,^1^ including newly confirmed cases, newly asymptomatic infections and newly asymptomatic infections converted to confirmed cases. Epidemiological survey data of positive infected cases included age, sex, place of residence, disease severity (asymptomatic/mild/moderate/severe/critical) and identification methods (community screening/centralized isolation screening/home quarantine screening/mass screening/risk region screening/close contact screening/others).

### Time-varying SQEIAHR model

In the study of dynamic models of infectious diseases, individuals are abstracted into several compartments: Susceptible (S), Exposed (E), Infected (I) and Removed (R), and transitions between individuals constitute different transmission models. The classical transmission models include SI, SIR, SIS and SEIR, of which SIR and SEIR occupy a central place in epidemiology (17–19). Given the existence of a latent period for COVID-19 and the ability of patients to acquire some immunity after healing, a modified SEIR model was selected for analysis in this study.

**Figure 1:**
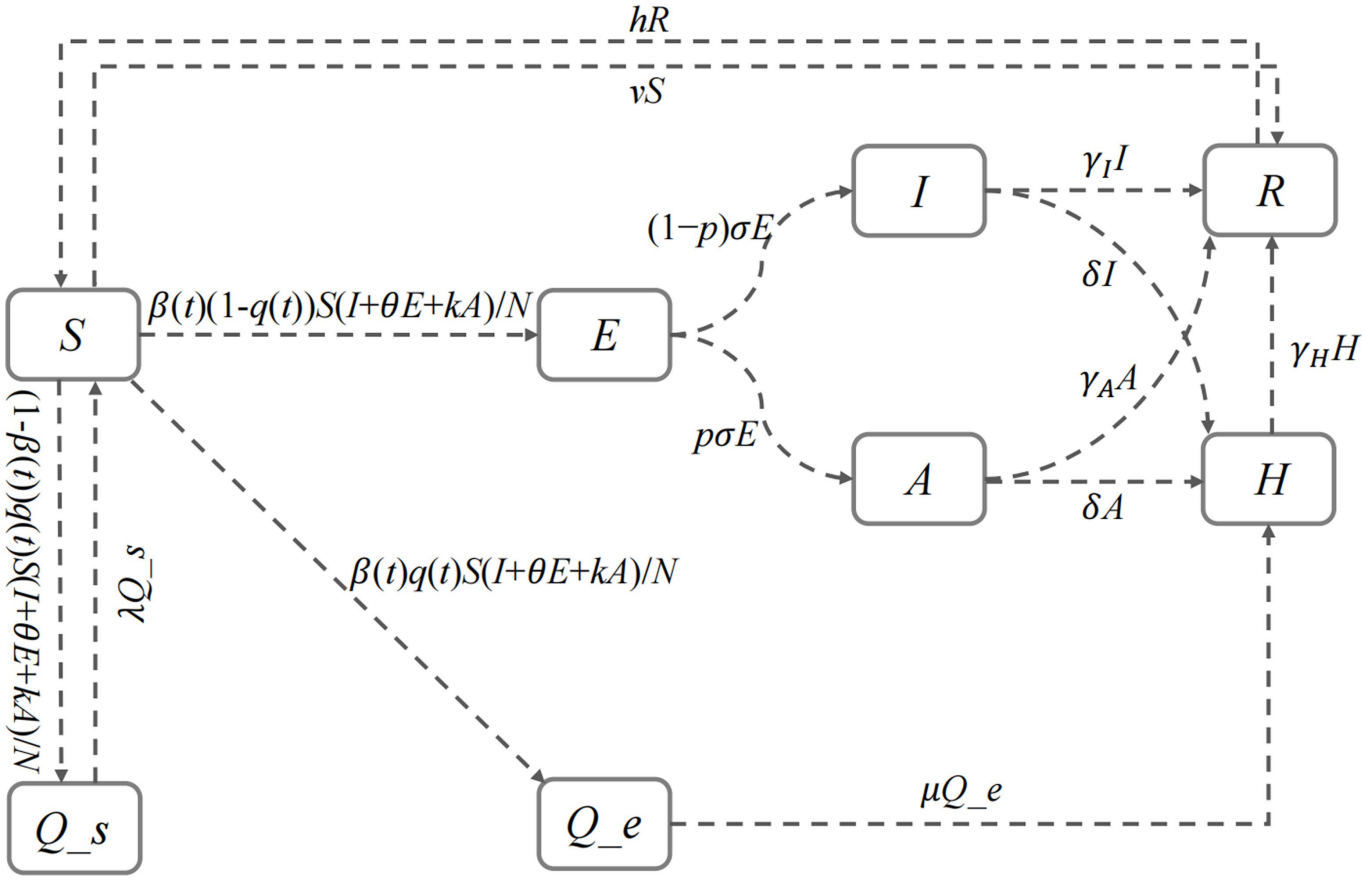
The structure of the SQEIAHR model.

Figure 1 provides a comprehensive description of the time-varying Susceptible-Quarantined Susceptible-Exposed-Quarantined Exposed-Infected-Asymptomatic-Hospitalized-Removed (SQEIAHR) model established under the actual epidemic characteristics in Hohhot, such as the large number of asymptomatic infections and the changing transmission rate. The model has eight compartments (*S*, *Q_s*, *E*, *Q_e*, *I*, *A*, *H*, *R*) and 14 parameters ( $\beta \left(t\right),q\left(t\right),\theta ,k,\lambda ,v,h,\sigma ,p,\delta ,\mu ,\gamma_{I},\gamma_{A},\gamma_{H}$ ), of which β(t) and q(t) are related to intervention strategies. It is important to note that compartment I did not include those who moved from an asymptomatic infection to a confirmed case during isolation.

In the SQEIAHR model, we have the constraint *N* = *S* + *Q_s* + *E* + *Q_e* + *I* + *A* + *H* + *R* with the following set of differential equations:

(1) $$\begin{array}{l} \frac{dS}{dt}=-\left[\beta \left(t\right)+\left(1-\beta \left(t\right)\right)q\left(t\right)\right]S\left(I+\theta E+kA\right)\\ \;\;\;\;\;\;\;\;\;\;\;\;\;\;/N+\lambda Q\_s-vS+hR, \end{array}$$

(2) $$\frac{dQ\_s}{dt}=\left(1-\beta \left(t\right)\right)q\left(t\right)S\left(I+\theta E+kA\right)/N-\lambda Q\_s,$$

(3) $$\frac{dE}{dt}=\beta \left(t\right)\left(1-q\left(t\right)\right)S\left(I+\theta E+kA\right)/N-\sigma E,$$

(4) $$\frac{dQ\_e}{dt}=\beta \left(t\right)q\left(t\right)S\left(I+\theta E+kA\right)/N-\mu Q\_e,$$

(5) $$\frac{dI}{dt}=\left(1-p\right)\sigma E-\left(\delta +\gamma_{I}\right)I,$$

(6) $$\frac{dA}{dt}=p\sigma E-\left(\delta +\gamma_{A}\right)A,$$

(7) $$\frac{dH}{dt}=\delta \left(I+A\right)+\mu Q\_e-\gamma_{H}H,$$

(8) $$\frac{dR}{dt}=\gamma_{I}I+\gamma_{A}A+\gamma_{H}H+vS-hR.$$

The compartments and parameters in the above equations are described in Table 1.

**Table 1:** Descriptions of the model compartments and parameters.

	Category	Description
Compartments	*S*	susceptible individuals
	*Q_s*	quarantined susceptible individuals
	*E*	exposed individuals with no symptoms who transmit the virus
	*Q_e*	quarantined exposed individuals
	*I*	confirmed cases that show typical clinical symptoms
	*A*	asymptomatic infections that do not show typical clinical symptoms or corresponding CT imaging manifestations
	*H*	hospitalized individuals undergoing treatment
	*R*	recovered individuals who are still at risk of becoming susceptible
Parameters	v	immunity threshold (vaccination rate × vaccine protection rate)
	p	proportion of asymptomatic infections among infected cases
	σ	incubation rate
	h	reduction rate of the antibody level
	λ	quarantine release rate
	β(t)	time-varying transmission rate
	q(t)	time-varying quarantine rate
	θ	infectivity coefficient of exposed individuals
	k	infectivity coefficient of asymptomatic infections
	δ	hospitalization rate of positive infected cases
	μ	hospitalization rate of quarantined exposed individuals
	$\gamma_{I}$	recovery rate of confirmed cases
	$\gamma_{A}$	recovery rate of asymptomatic infections
	$\gamma_{H}$	recovery rate of hospitalized individuals

In this study, β(t) and q(t) are piecewise functions that represent the time-varying transmission rate and time-varying quarantine rate, respectively. The function expression was split into two segments using October 3, 2022, as the turning point. Since October 3, 2022, Hohhot has upgraded its level of management and control, applied more rigorous measures and paid greater attention to the hardest-hit and high-risk areas. In Eq. (9), $\beta_{0}$ represents the initial transmission rate, w stands for the exponential decline rate of the transmission rate after taking preventive and control measures, and $t_{1}$ refers to October 3, 2022. In Eq. (10), $q_{0}$ and $q_{1}$ represent the initial quarantine rate and the maximum quarantine rate after the implementation of preventive and control measures, respectively, r stands for the exponential increase rate of the quarantine rate, and $t_{1}$ refers to October 3, 2022.

(9) $$\beta \left(t\right)=\{\begin{array}{cc} \beta_{0} & t<t_{1},\\ \beta_{0}\times e^{-w\left(t-t_{1}\right)} & t\geq t_{1}. \end{array}$$

(10) $$q\left(t\right)=\{\begin{array}{cc} q_{0} & t<t_{1},\\ \left(q_{0}-q_{1}\right)\times e^{-r\left(t-t_{1}\right)}+q_{1} & t\geq t_{1}. \end{array}$$

The transmission process of COVID-19 in the SQEIAHR model is as follows:

The model assumes that *S* enters *E*, *Q_s*, and *Q_e* with probabilities β(t)(1−q(t)) , (1−β(t))q(t) , and β(t)q(t) after exposure to infectious sources such as *E*, *I*, and *A*. If *S* acquires specific immunity through vaccination, then it will enter *R* with probability v . If no abnormality is found at the end of the quarantine period 1/λ , then *Q_s* will re-enter *S*. *E* enters *A* and *I* at rates pσ and (1−p)σ . *I*, *A*, and *Q_e* enter *H* for treatment at rates δ , δ , and μ. The cured *I*, *A*, and *H* enter *R* at rates $\gamma_{I}$ , $\gamma_{A}$ , and $\gamma_{H}$ . Regarding *R*, as the antibody level decreases, it re-enters *S* at rate h .

### Estimation of model parameters

The Markov Chain Monte Carlo (MCMC) method has become very popular for Bayesian computation in complex statistical models. The basic idea is to first construct a Markov Chain whose desired distribution is close to its equilibrium distribution, and then generate samples of the posterior distribution through this Markov Chain, and finally perform Monte Carlo integration based on the valid samples when the Markov Chain reaches its equilibrium. To compensate for the low acceptance probability of MCMC sampling, we used the Metropolis-Hastings (M-H) algorithm to sample the posterior distribution of $\beta_{0},w,q_{0},q_{1},r,\theta ,k,\delta ,\mu ,\gamma_{I},\gamma_{A},\gamma_{H}\;$ (20, 21). We ran this algorithm for 60,000 iterations with a burn-in period of 55,000. The posterior means and 95% Bayesian credible intervals for 12 parameters are displayed in Table 2, and the model compartments and remaining parameters were derived from actual epidemic or literature reports.

**Table 2:** The values and sources of model compartments and parameters.

Compartments	Value	Source	Parameters	Value	Source
*S*(0)	3,495,944	Actual epidemic	v	0.699	Actual epidemic
*Q_s*(0)	0	Actual epidemic	p	0.851	Actual epidemic
*E*(0)	54	Actual epidemic	σ	1/4	Actual epidemic
*Q_e*(0)	0	Actual epidemic	h	0.730	Literature reports (22)
*I*(0)	1	Actual epidemic	λ	1/14	Literature reports (23)
*A*(0)	0	Actual epidemic	$\beta_{0}$	1.500 (1.496 ~ 1.503)	MCMC
*H*(0)	1	Actual epidemic	w	1.001 (0.998 ~ 1.004)	MCMC
*R*(0)	0	Actual epidemic	$q_{0}$	0.085 (0.081 ~ 0.088)	MCMC
			$q_{1}$	0.499 (0.496 ~ 0.503)	MCMC
			*r*	0.999 (0.996 ~ 1.003)	MCMC
			θ	1.000 (0.996 ~ 1.003)	MCMC
			k	1.000 (0.996 ~ 1.004)	MCMC
			δ	0.801 (0.798 ~ 0.804)	MCMC
			μ	0.799 (0.796 ~ 0.803)	MCMC
			$\gamma_{I}$	0.101 (0.096 ~ 0.104)	MCMC
			$\gamma_{A}$	0.100 (0.096 ~ 0.103)	MCMC
			$\gamma_{H}$	0.100 (0.096 ~ 0.103)	MCMC

### Time-varying reproduction number of disease-free equilibrium

The basic reproduction number (*R*_0_) is defined as the number of second-generation cases caused by an individual infected in a fully susceptible population without any intervention. It is a threshold indicator for measuring the transmission capacity of infectious diseases and determining the point of disease-free equilibrium. Notably, the effective reproduction number (*R*_e_) is more appropriate than *R*_0_ for assessing the effectiveness of vaccines or other non-pharmaceutical interventions. It can be used to track changes in the reproduction number, and whether the epidemic is controlled depends on whether *R*_e_ is consistently less than 1.

The next-generation matrix method (24, 25) was applied to calculate *R*_e_:

(11) $$R_{e}=\beta \left(t\right)\left(1-q\left(t\right)\right)\left[\frac{\theta }{\sigma }+\frac{1-p}{\delta +\gamma_{I}}+\frac{kp}{\delta +\gamma_{A}}\right]$$

### Statistical analysis

The calculations of the SQEIAHR compartmental model were performed using Python software (version 3.7.1, Python Software Foundation, Python Language Reference). The figures were produced by GraphPad Prism software (version 9.0, La Jolla, CA, United States). The coefficient of determination (*R*^2^) was used to evaluate the goodness-of-fit of the model.

## Results

### Epidemiological characteristics of COVID-19

As of October 18, 2022, a total of 4,889 positive infected cases have been reported in Hohhot. Among them, 4,160 cases were asymptomatic, 727 were mild, 2 were moderate, and there were no severe or critical cases.

### Spatiotemporal distribution

**Figure 2:**
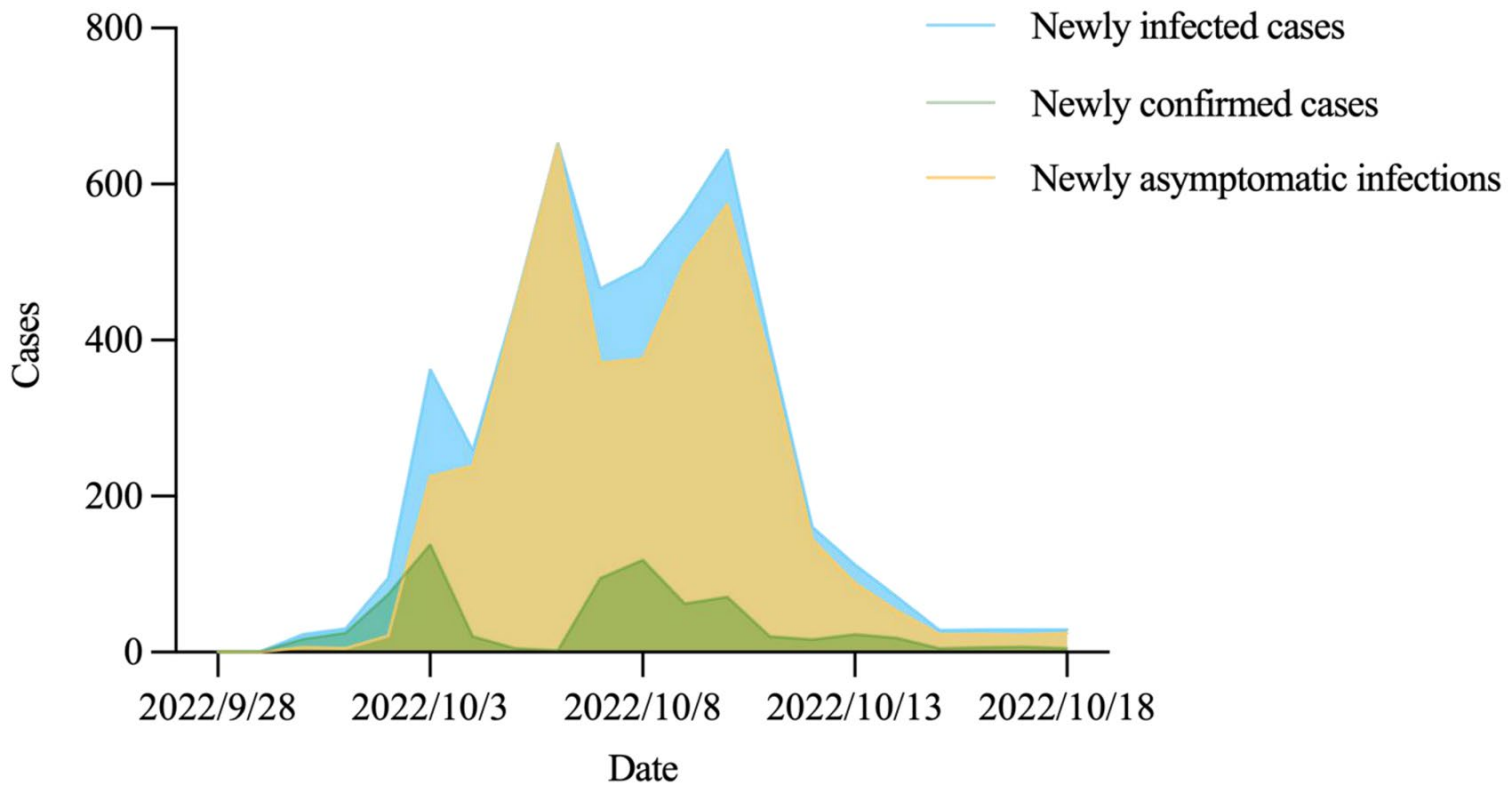
The daily number of new infections in Hohhot.

**Figure 3:**
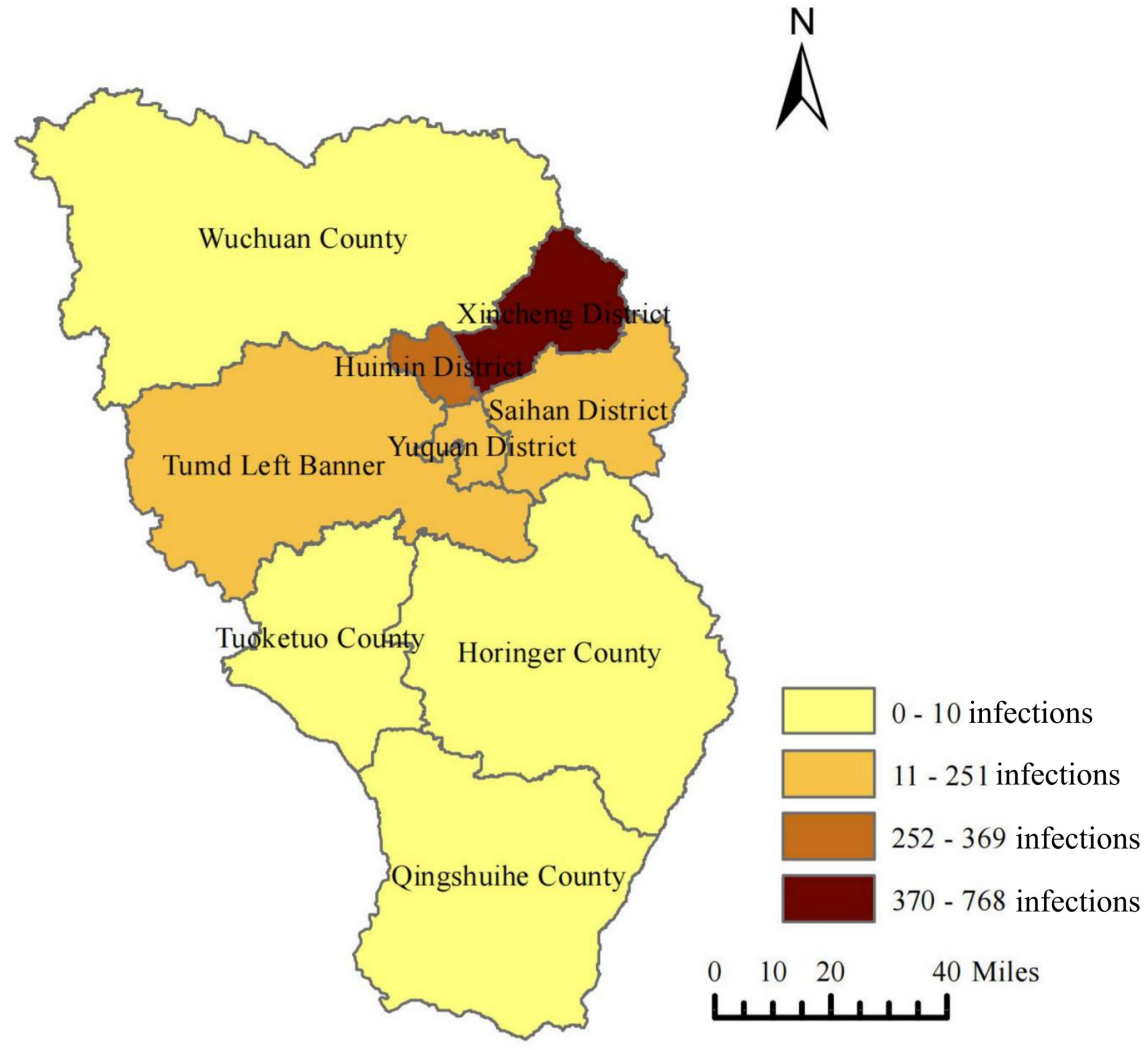
The spatial distribution of COVID-19 cases in Hohhot.

From September 28, 2022, the daily number of new infections showed a fluctuating growth trend, reaching the first peak (*n* = 653) on October 6, 2022, and then rapidly decreasing after arriving at the second peak (*n* = 645) on October 10, 2022 (Figure 2). A spatial distribution map of cumulative infected cases in Hohhot is shown in Figure 3. Among the information officially released by the Hohhot government on the activity trajectories of the 1,941 indigenous positive infected cases, nearly half of the cases were found in Xincheng District, followed by Huimin, Yuquan, and some other nearby districts, while southern areas such as Tuoketuo, Helingeer and Wuchuan had extremely low infection rates, accounting for a total of 0.72%.

### Sociodemographic distribution

**Figure 4:**
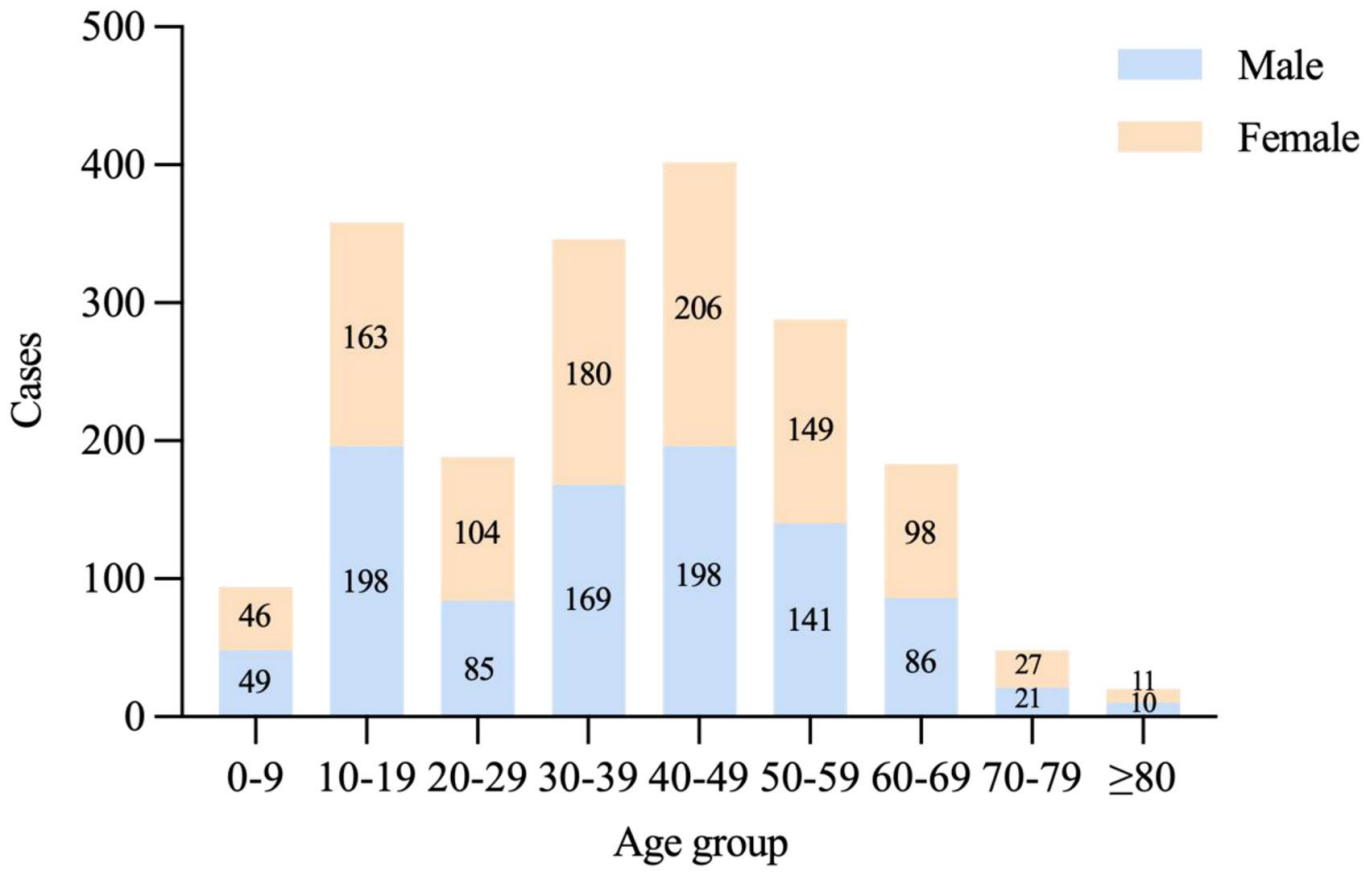
The distribution of age and sex of positive infected cases in Hohhot.

**Figure 5:**
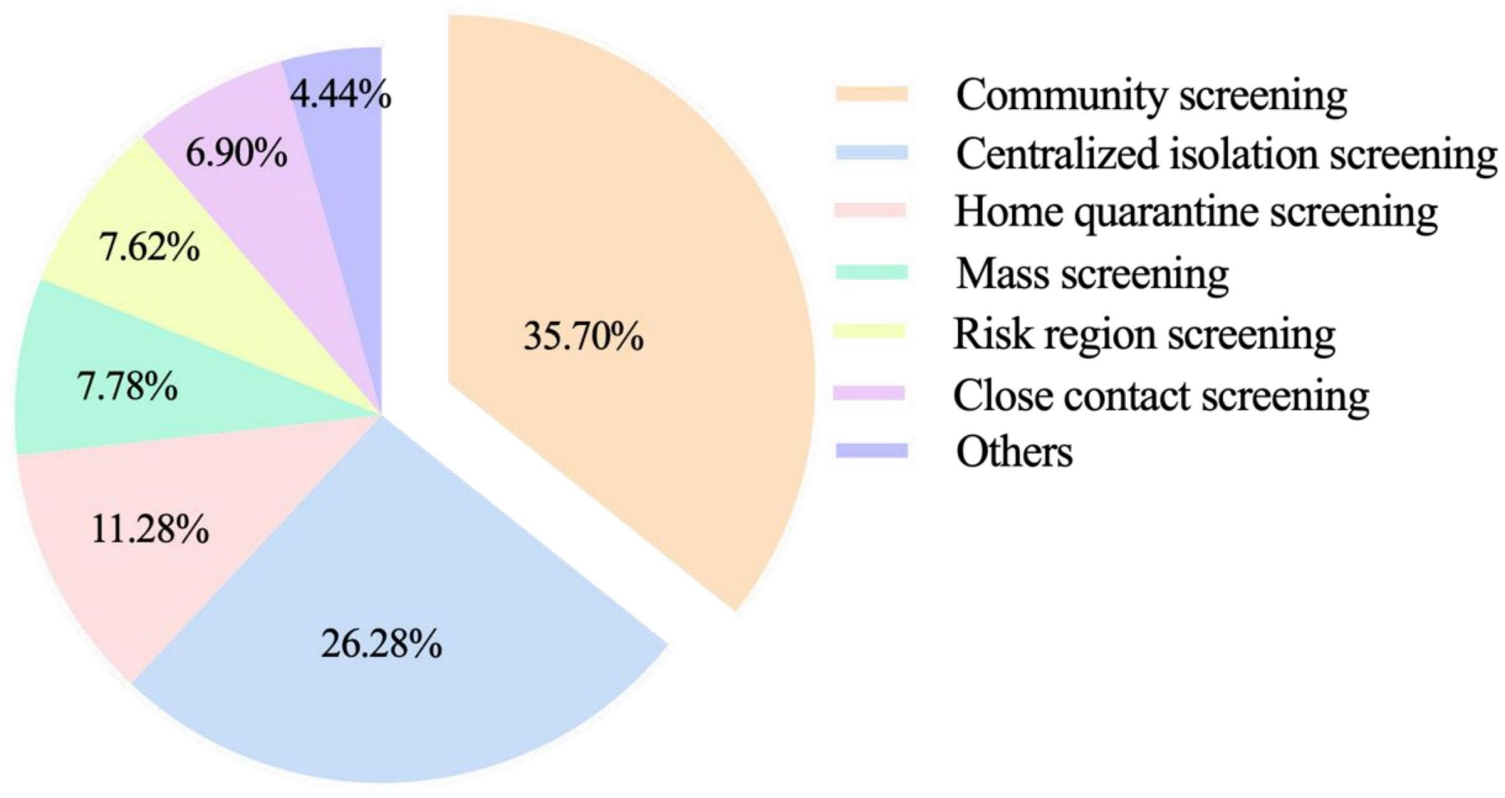
The distribution of identification methods of positive infected cases in Hohhot.

People with COVID-19 had broad age-specific variation, ranging from 3 days to 89 years of age. People in the 30–59 age group were the main victims of the current outbreak, accounting for 53.74% of the total cases. In terms of sex, females (*n* = 984) and males (*n* = 957) were almost equally affected, and the sex ratio was approximately 1.03:1 (Figure 4). In addition, community screening (35.70%) and centralized isolation screening (26.28%) were the main ways to detect cases, suggesting that the focus should be on the social transmission of the epidemic (Figure 5).

### Transmission dynamics of COVID-19

#### Prediction of COVID-19 cases

**Figure 6:**
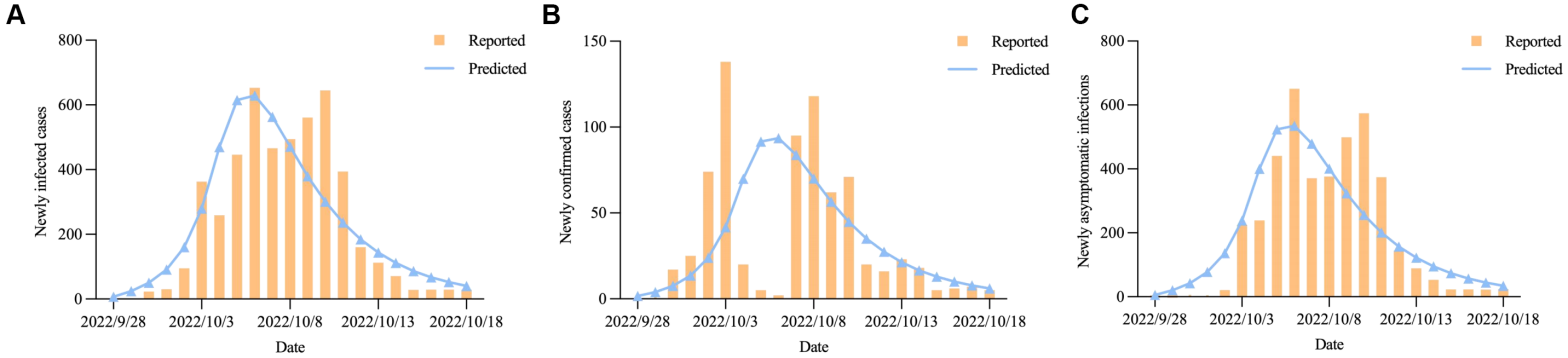
The predicted daily number of new COVID-19 cases in Hohhot; **(A)** Newly infected cases; **(B)** Newly confirmed cases. **(C)** Newly asymptomatic infections.

During the epidemic, the four most important indicators were the time to reach the peak, the time to achieve dynamic zero-COVID, the number of peak cases, and the cumulative number of infections (26). We plotted the actual daily number of new infections up to October 18, 2022, onto our forecasted curve and found that there was an overall good fit between our projected and reported data (*R*^2^ = 0.739, *p* < 0.001) (Figure 6A). The number of newly confirmed cases and newly asymptomatic infections was expected to reach an inflection point on October 6, 2022 (*n* = 629), with the sum of the two dropping below 100 cases on October 15, 2022, after which the epidemic gradually died out (Figures 6B,C). Our model predicted that the final affected population in Hohhot would reach 4,963 (95% confidential interval (95%CI): 4,692 ~ 5,267), including 740 (95%CI: 699 ~ 786) confirmed cases and 4,224 (95%CI: 3,993 ~ 4,481) asymptomatic infections (Table 3). The above projections demonstrated the reliability of our model in assessing COVID-19 trends (actual peak date: October 6, 2022; actual dynamic zero-COVID date: October 14, 2022; actual number of peak cases: 653; actual cumulative number of infections: 4,889).

**Table 3:** The predicted cumulative number of COVID-19 cases in Hohhot in different time periods.

Time periods	Cumulative infected cases	Cumulative confirmed cases	Cumulative asymptomatic infections
9-28-2022 to 10-3-2022	613 (592 ~ 636)	92 (89 ~ 96)	521 (503 ~ 540)
9-28-2022 to 10-8-2022	3,361 (3,189 ~ 3,553)	501 (476 ~ 530)	2,859 (2,713 ~ 3,022)
9-28-2022 to 10-13-2022	4,605 (4,356 ~ 4,884)	686 (649 ~ 729)	3,919 (3,707 ~ 4,155)
9-28-2022 to 10-18-2022	4,963 (4,692 ~ 5,267)	740 (699 ~ 786)	4,224 (3,993 ~ 4,481)

#### Prediction of the effective reproduction number

**Figure 7:**
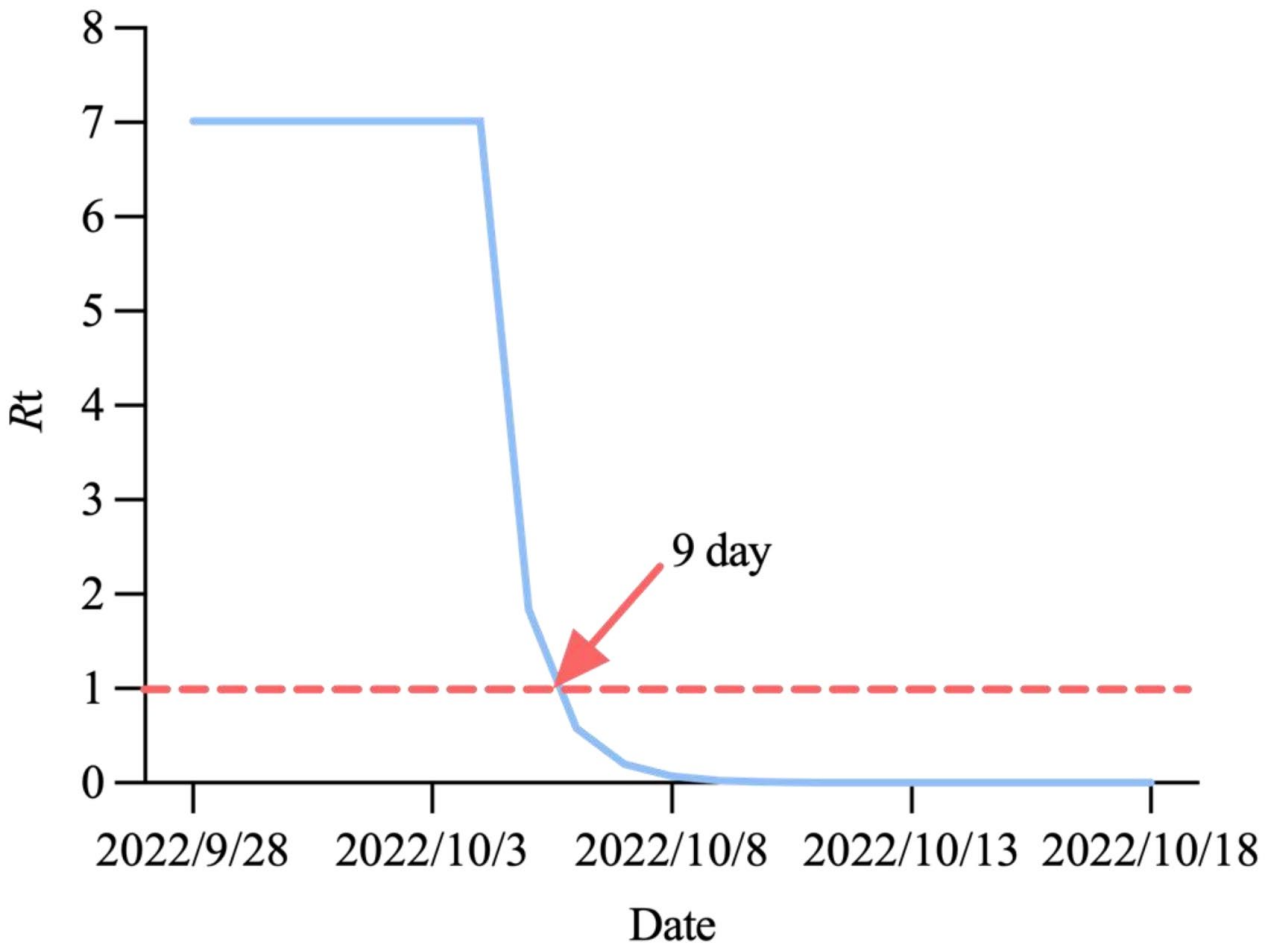
Change trend of R_e_ in Hohhot.

The effective reproduction number can be discussed in two stages. During the first stage, from September 28, 2022, to October 3, 2022, the risk of virus transmission increased as population mobility increased during the National Day holiday, with an *R*_0_ of approximately 7.01 (95%CI: 6.93 ~ 7.09). The second stage occurred after October 4, 2022. Following the implementation of a series of measures, such as multiple rounds of nucleic acid testing and resident travel control, *R*_e_ began to decline rapidly until it dropped below 1.0 on October 6, 2022, and has since maintained a steady trend (Figure 7).

### Scenario analysis of higher stringency measures

**Figure 8:**
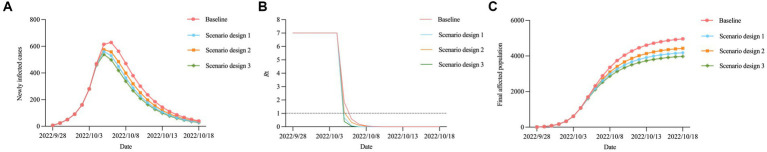
Trends in the epidemic under different measure scenarios. **(A)** Newly infected cases. **(B)** R_e_. **(C)** final affected population.

In this study, higher stringency measures were aimed at curbing the spread of the epidemic by decreasing the transmission rate and increasing the quarantine rate. The scenario design of higher stringency measures was divided into three parts: (1) increasing the exponential decline rate of the transmission rate (2.000 vs. 1.001), (2) increasing the maximum quarantine rate (0.700 vs. 0.499) and the exponential increase rate of the quarantine rate (2.000 vs. 0.999), and (3) increasing the exponential decline rate of the transmission rate (2.000 vs. 1.001), the maximum quarantine rate (0.700 vs. 0.499), and the exponential increase rate of the quarantine rate (2.000 vs. 0.999). Our results showed that scenario design 3 was the optimal strategy: the time to reach the peak, the time to achieve dynamic zero-COVID, and the time for the *R*_e_ curve to fall to 1.0 would be brought forward to October 5, 2022, October 13, 2022, and October 5, 2022, respectively, while the number of peak cases and final affected population would be greatly reduced by 14.31% and 19.83%, respectively (Figures 8A–C; Table 4).

**Table 4:** Values of epidemic indicators under different measure scenarios.

Category	Parameter (value)	Time to peak	Time to dynamic zero-COVID	Time to *R*_e_ below 1.0	Peak cases	Final affected population
Baseline	w (1.001)	10-6-2022	10-15-2022	10-6-2022	629	4,963
	$q_{1}$ (0.499)					
	r (0.999)					
Scenario design 1	w (2.000)	10-5-2022	10-14-2022	10-5-2022	561	4,181
	$q_{1}$ (0.499)					
	r (0.999)					
Scenario design 2	w (1.001)	10-5-2022	10-14-2022	10-6-2022	575	4,434
	$q_{1}$ (0.700)					
	r (2.000)					
Scenario design 3	w (2.000)	10-5-2022	10-13-2022	10-5-2022	539	3,979
	$q_{1}$ (0.700)					
	r (2.000)					

## Discussion

Overall, the peak of the epidemic in Hohhot has passed, and the number of new cases is steadily declining, indicating that the epidemic has been effectively controlled. The time-varying SQEIAHR compartmental model we proposed in this study captured some important qualitative features and hence could provide guidance in policy-making. To the best of our knowledge, this is the most comprehensive report investigating the latest epidemiological characteristics and development trends of COVID-19 in Hohhot.

According to epidemiological investigations, the majority of cases in the current outbreak were asymptomatic and mild, which may be related to the fact that COVID-19 vaccination reduces the threat of serious illness and death (27). People aged 30–59 years were at particular risk of COVID-19, contrary to the finding that older people were the main vulnerable group (28). We conjectured that this might be because the middle-aged population has a wider range of activities and is more likely to be exposed to the virus, for example, engaging in group work or recreation. The key to stopping the spread of the virus is to identify all infections quickly. In Hohhot, those who had positive nucleic acid test results were mainly derived from community screening, indicating that the efficiency and scope of nucleic acid testing should be further improved (29). Wu Zunyou, chief epidemiologist at the Chinese Center for Disease Control and Prevention, recommended that “each round of mass screening should be completed within one to three days and be guaranteed to cover all target groups.” Additionally, areas with a large number of cases were primarily concentrated in the central part of Hohhot, and therefore, more medical resources should be prepared in advance and allocated to Xincheng and surrounding districts as soon as possible.

Dynamic model is an important method for the theoretical and quantitative study of infectious diseases. It can be used to describe the pattern of disease transmission, predict disease status and assess the effectiveness of prevention and control measures (30–32). The time-varying SQEIAHR model we constructed is methodologically robust and built on the classical SEIR model previously applied to forecast the development trends of infectious diseases. In earlier studies, scholars did not consider the impact of asymptomatic infections, immune threshold, and antibody titer reduction on the spread of the epidemic in their modeling due to an inadequate understanding of the transmission mechanisms and clinical characteristics of COVID-19 (33, 34). Our SQEIAHR model was inspired by the key factors mentioned above and accommodated the influence of the time-varying transmission rate, time-varying quarantine rate and reinfection, so the prediction results were highly robust.

The four key indicators predicted by our model were (1) the epidemic peak on October 6, 2022, (2) dynamic zero-COVID on October 15, 2022, (3) the number of peak cases of 629, and (4) the cumulative number of infections of 4,963, all of which were in fairly good agreement with the actual situation in Hohhot. Compared to the outbreak also caused by Omicron BF.7, the number of infections in Beijing far exceeded that in Hohhot (35). The reason for this is that at the time of the outbreak in Hohhot, China was still employing the “dynamic zero-COVID policy,” whereas the outbreak in Beijing occurred during the transition stage from the announcement of 20 measures to the gradual liberalization of the epidemic, so the intervention intensity was much more lenient than before, leading to a surge in infections. The effective reproduction number is a critical threshold parameter in epidemiology that can be used to measure the real-time transmissibility of an outbreak (36). Initially, *R*_0_ was a constant greater than 7. After the implementation of a series of control measures, *R*_e_ showed a rapid downward trend, and it took only nine days from the discovery of the first case to achieve an *R*_e_ below 1.0. After the outbreak of COVID-19 in Shanghai on March 1, 2022, *R*_e_ did not drop below 1.0 until day 45 (37). This reflects the remarkable achievement of epidemic prevention and control efforts in Hohhot.

Non-pharmaceutical interventions such as decreasing the transmission rate and increasing the quarantine rate have been recommended in the battle against COVID-19. We conducted a quantitative comparison of different intervention strategies and found significant effects of a combination of both in shortening the time to peak, the time to dynamic zero-COVID and the time to an *R*_e_ below 1.0, while substantially reducing the number of peak cases and accumulated cases. These results emphasized the importance of decreasing the transmission rate by travel restrictions and the necessity of increasing the quarantine rate by close-contact tracing. Liu W et al. also recommended that isolation measures be implemented in communities and outbreak sites in a timely manner to ensure social distancing between people, thereby reducing the level of human contact (38). It is worth noting that the values of β(t) and q(t) in the scenario design only increased by a very small amount, and we have reason to believe that with the further strengthening of prevention and control measures, even better results will be achieved.

### Limitations

Admittedly, this study has some limitations. First, our model did not take into account the difference between the rate of reinfection among recovered individuals and the rate of infection in the general population. Second, in addition to decreasing the transmission rate and increasing the quarantine rate, other interventions, such as promoting a social consensus on self-protection and expanding environmental decontamination, may also have a potential impact on the development of the epidemic. In reality, the end of the outbreak may come earlier than we predicted. In future work, efforts must be made to optimize the SQEIAHR model by introducing additional parameters that can reflect different infection rates and non-pharmaceutical interventions. Work is also needed to test the model in various geographical and demographic contexts.

## Conclusion

In this paper, we developed a time-varying SQEIAHR compartmental model to investigate the transmission dynamics of COVID-19. Our projections suggested that this model was well suited to capture key epidemic indicators, including the time to reach the peak, the time to achieve dynamic zero-COVID, the number of peak cases, and the cumulative number of infections. Moreover, decreasing the transmission rate by travel restrictions and increasing the quarantine rate by close-contact tracing could achieve remarkable results in curbing the spread of COVID-19. These findings can not only help health departments prepare in advance for a possible outbreak of COVID-19 but also provide an important reference for optimizing non-pharmaceutical intervention programs.

## Data availability statement

The original contributions presented in the study are included in the article/Supplementary material, further inquiries can be directed to the corresponding authors.

## Author contributions

JX, HY, and TW conceived the study and revised the manuscript critically. JL, LL, and LH collected the data. YM prepared the first draft of the manuscript. YM, SX, and YL developed the model and analyzed the results. SX and YQ prepared the tables and figures. All authors contributed to the article and approved the submitted version.

## Funding

This study was supported by the National Key Research and Development Program of China (2021YFC2301603), the Major Science and Technology Project of Shanxi Province (202102130501003 and 202005D121008), and the Special Foundation on COVID-19 of Shanxi Health Commission (16).

## Conflict of interest

The authors declare that the research was conducted in the absence of any commercial or financial relationships that could be construed as a potential conflict of interest.

## Publisher’s note

All claims expressed in this article are solely those of the authors and do not necessarily represent those of their affiliated organizations, or those of the publisher, the editors and the reviewers. Any product that may be evaluated in this article, or claim that may be made by its manufacturer, is not guaranteed or endorsed by the publisher.

## Footnotes

^1^http://wjw.nmg.gov.cn/.
